# Does the Consumption of Metformin Correlate With a Reduction in Mortality Among Patients With Type 2 Diabetes and COVID-19 in Morocco?

**DOI:** 10.7759/cureus.77288

**Published:** 2025-01-11

**Authors:** Bouchra Benfathallah, Abha Cherkani Hassani, Samia El Hilali, Redouane Abouqal, Laïla Benchekroun

**Affiliations:** 1 Laboratory of Biochemistry and Molecular Biology, Faculty of Medicine and Pharmacy of Rabat, Mohammed V University, Rabat, MAR; 2 Laboratory of Analytical Chemistry and Bromatology, Faculty of Medicine and Pharmacy of Rabat, Mohammed V University, Rabat, MAR; 3 Laboratory of Biostatistics, Clinical Research and Epidemiology and Department of Public Health, Faculty of Medicine and Pharmacy of Rabat, Mohammed V University, Rabat, MAR; 4 Laboratory of Community Health and Department of Public Health, Faculty of Medicine and Pharmacy of Rabat, Mohammed V University, Rabat, MAR; 5 Laboratory of Biostatistics, Clinical Research and Epidemiology, Faculty of Medicine and Pharmacy of Rabat, Mohammed V University, Rabat, MAR; 6 Central Laboratory of Biochemistry, Ibn Sina University Hospital, Faculty of Medicine and Pharmacy of Rabat, Mohammed V University, Rabat, MAR

**Keywords:** covid-19, metformin, morocco, mortality, type 2 diabetes

## Abstract

Objectives: To assess whether metformin therapy for type 2 diabetes (T2DM) was associated with a reduced mortality rate in patients hospitalized for COVID-19 compared to other antihyperglycemic drugs.

Methods: This retrospective study included patients with T2DM who tested positive for SARS-CoV-2 between 1 August 2020 and 1 August 2021. The patients were required to be aged over 18 years old and to be undergoing treatment for hyperglycemia, whether with metformin, other oral antidiabetic drugs, or insulin. A data exploitation sheet was completed for each patient. The Jamovi (https://www.*jamovi*.org/) software was applied to conduct the statistical analyses. Multivariate logistic regression was used to determine whether metformin use was associated with reduced mortality among patients with T2DM and COVID-19.

Results: We identified 115 COVID-19 patients with T2DM, of whom 41 were on metformin, 35 patients were on insulin, and 39 patients were on other oral antihyperglycemic agents; the average age of patients was 65.5±13.2 years, and 52.2% were male. The mortality rate was lower in the metformin user group (21.1%) compared to the non-user group (78.9%). The multivariate logistic regression model indicated that age (OR=1.06; 95% CI (1.02-1.10); p=0.002) and glycemia (OR=1.49; 95% CI (1.05-2.11); p=0.024) were significantly associated with mortality in patients with T2DM and COVID-19. Whereas, the use of metformin was identified as a protective factor (OR=0.34 95% CI (0.12-0.95); p=0.041).

Conclusion: This study highlighted that metformin seems to be associated with significantly decreased mortality in adults with T2DM and COVID-19.

## Introduction

The pandemic caused by the SARS-CoV-2 virus constituted a significant threat to global human health, resulting in a particularly severe form of the disease and poorer clinical outcomes in diabetic patients compared to their non-diabetic counterparts [1]. Several studies have reported that hyperglycemia or typical complications of diabetes increase the severity and mortality of COVID-19 [2-4]. Other studies have focused on antihyperglycemic treatments for individuals with type 2 diabetes during SARS-CoV-2 infection, with a particular emphasis on the impact of these treatments on the severity of SARS-CoV-2 infection and mortality in this patient population [5-8].

Metformin is one of the most commonly utilized antidiabetic medications. Given its pervasive usage, the influence of metformin therapy on the progression of SARS-CoV-2 infection is of paramount importance. The use of metformin has been associated with a reduction in mortality among patients with type 2 diabetes mellitus (T2DM) who have been diagnosed with COVID-19 [6,9,10]. In the CORONADO trial, which assessed the efficacy of various antidiabetic medications, metformin treatment demonstrated the most favorable outcome in reducing mortality [11]. A retrospective study conducted in the Tongji Hospital of Wuhan, China from January 2020 to March 2020 by Luo and colleagues examined the effects of metformin on 283 patients with diabetes, of whom 104 received metformin and 179 did not. They observed a significantly lower in-hospital mortality in the former group compared to the latter [12]. A multicenter retrospective study corroborated this finding, indicating that metformin treatment before hospital admission was associated with a reduced likelihood of requiring intensive care unit admission, with a dose-dependent correlation [13].

In contrast, the use of metformin has been demonstrated to induce acidosis in diabetic patients infected with SARS-CoV-2, yet it has not been shown to increase mortality; Consequently, metformin treatment was initially contraindicated for patients with both diabetes mellitus and SARS-CoV-2 infection [14]. Nevertheless, subsequent research has indicated the potential advantages of this approach for the management of diabetes [15]. Of note, metformin remains contraindicated in patients with an elevated risk of developing acidosis [16].

While international studies have explored the relationship between metformin use and COVID-19 mortality, no such investigation has been conducted in Morocco, where the healthcare context may yield unique findings. In this regard, the current study aims to evaluate the impact of metformin, in comparison with alternative antihyperglycemic drugs, on the in-hospital mortality among type 2 diabetic patients with SARS-CoV-2.

## Materials and methods

Type and population of the study

This is a retrospective cohort, descriptive, comparative, and analytical study of the medical records of patients with T2DM who were admitted and hospitalized with COVID-19 in the Acute Medical Unit (AMU) of Avicenne Hospital in Rabat, between August 1, 2020, and August 1, 2021.

Study eligibility criteria

The study population comprised adult patients (>18 years old) with T2DM. Their primary treatment was metformin, insulin, or another oral antihyperglycemic drug. They were subsequently admitted to the AMU of Avicenne Hospital in Rabat with a confirmed SARS-CoV-2 infection, as determined by RT-PCR and/or thoracic computed tomography (CT) using the CO-RADS classification system.

Concerning the exclusion criteria, all patient files that were unsuitable for inclusion were excluded from the study (these comprised pregnant women, patients under the age of 18 years, and files with missing data).

Disease severity was defined by the World Health Organization (WHO) guidelines. Patients with non-severe COVID-19 did not require supplemental oxygen. These patients exhibited pneumonia but no indications of severe pneumonia. Patients with severe SARS-CoV-2 infection exhibited respiratory distress, a respiratory rate exceeding 30 breaths per minute, or a SpO_2_ level of less than 93% in ambient air [17]. Mortality was defined as in-hospital death due to COVID-19.

Procedure

A convenience cluster sampling method was used. The patients were divided into two groups based on the diabetes treatment they received during their hospitalization. The initial cohort was treated with metformin, while the second cohort was administered other oral antihyperglycemic drugs. To balance blood glucose levels, insulin was administered to all patients post-admission for a limited period. The mean blood glucose level during the hospitalization period was calculated from the data extracted from the patient files.

Data collection 

A data sheet was created for each patient, detailing the following information: socio-demographic characteristics (age, sex, and origin); medical history and co-morbidities including hypertension and cardiovascular disease; pre-admission diabetes treatment; clinical characteristics such as oxygen saturation and heart rate; biological and imaging findings (including hemoglobin, leukocyte count, D-dimer, blood glucose, urea, and creatinine levels); duration of hospital stay; therapeutic management; and the progression of SARS-CoV-2 infection.

Statistical analysis

The data were entered using the Sphinx Plus² software. The statistical processing and analysis were conducted using the Jamovi software, version 2.6.2 (https://www.jamovi.org/). The initial step involved the production of descriptive statistics, which were employed to represent the variables under investigation. Continuous variables were presented as either means and standard deviations or medians and interquartile ranges, as appropriate. Category variables were presented as total numbers and percentages.

The associations between the variables were determined using the Chi-square test for qualitative variables, the Mann-Whitney test for quantitative variables with an asymmetric distribution, and the Student's t-test for symmetric distributions to compare two independent groups. Subsequently, simple and multiple logistic regression was employed to assess the risk factors associated with mortality among patients with T2DM and COVID-19. A p-value of less than 0.05 was considered statistically significant.

## Results

Demographic and clinical characteristics of the study population

The present study involved 115 patients with T2DM who had tested positive for SARS-CoV-2. Our findings revealed that 41 patients (35.7%) were on metformin before hospitalization, 35 patients (30.4%) were on insulin, and 39 patients (33.9%) were on other oral antihyperglycemic agents. The mean age of patients was 65.5±13.2 years, with a predominance of the 60-75 age group. Additionally, males accounted for 52.2% (n=60) of the total population. Furthermore, 97.4% (n=111) of patients resided in urban areas. The median time from the onset of symptoms to hospital admission was seven days, with approximately 50% of the study population having a hospital stay of nine days. Over half of the patients presented with symptoms including fever, cough, fatigue, and dyspnea.

The most prevalent comorbidity among patients with T2DM and a positive diagnosis of SARS-CoV-2 infection was hypertension, affecting 51.3% of the cohort (n=59). The CT imaging report indicated a significant discrepancy in lung involvement between patients, with a frequency of 37.9% (n=33) for CO-RADS class 5 (50-75% lung involvement). The recovery rate for the patients was 62.6% (n=72), while 38.3% (n=44) were admitted to the intensive care unit. The severity of the patient's conditions was observed in 75% (n=72) of the study population, and 33.0% (n=38) died. Table 1 summarizes the demographic and clinical characteristics of the study population.

**Table 1:** Demographics and clinical characteristics of the study participants *Data are expressed as n (%) **Median and IQR ***Mean and standard deviation The lung involvement rate was classified as follows: Class I (<25%), Class II (25-50%), Class III (50-75%), and Class IV (>75%).

Variables	Population
	N=115
Gender*	
Male	60 (52.2)
Female	55 (47.8)
Age* (Year) (n=114)	
<60	34 (29.8)
60-75	52 (45.6)
>75	28 (24.6)
Residence* (n=114)	
Urban	111 (97.4)
Rural	3 (2.6)
Duration from onset of symptoms to hospital admission (days)**	7 (5,10)
Presence of other comorbidities*	
Hyperblood pressure (HBP)	59 (51.3)
Cardiovascular disease	25 (21.7)
Inaugural diabetes	10 (8.7)
Chronic pulmonary disease	9 (7.8)
Chronic kidney disease	10 (8.7)
Other diseases	28 (24.3)
Preadmission diabetes treatment	
Metformin	41 (35.7)
Insulin	35 (30.4)
Other oral antidiabetic drugs	39 (33.9)
Presence of signs and symptoms*	
Fever	72 (77.4)
Cough	51 (60.0)
Asthenia	60 (52.2)
Myalgia	32 (34.4)
Dyspnea	60 (70.6)
Headache	16 (43.2)
Anorexia	13 (11.3)
Diarrhea	13 (50.0)
Vomiting	11 (9.6)
Oxygen saturation***	84±13.1
CT or (scanner)* (n=89)	
Class I	24 (27.6)
Class II	24 (27.6)
Class III	33 (37.9)
Class IV	6 (6.9)
Duration of hospitalization (days)**	9 (6,12)
Complication and evolution*	
Recovery	
Yes	72 (62.6)
No	43 (37.4)
Intensive care unit	
Yes	44 (38.3)
No	71 (61.7)
Death	
Yes	38 (33.0)
No	77 (67.0)
Severity	
Yes	72 (75.0)
No	24 (25.0)

Comparison of sociodemographic and clinical factors between metformin users and non-users

A comparative analysis examined the association of significant clinical and demographic outcomes between patients who had utilized metformin and those who had not. As illustrated in Table 2, there was no statistically significant difference in several variables, including gender, age, comorbidities, and symptoms. However, there were statistically significant differences in the frequency of recovery and transfer to the intensive care unit between the two groups, with p-values of 0.011 and 0.023, respectively. The mortality rate was lower in the metformin user group (21.1%) compared to the non-user group (78.9%), with a statistically significant p-value of 0.022.

**Table 2:** Sociodemographic and clinical factors associated with users of metformin and non-users *Missing data; lung involvement rate: Class I <25%, Class II 25-50%, Class III 50-75%, Class IV >75%.

Variable	Metformin	Other antihyperglycemic, N (%)	P-value
	N (%)		
Gender*			0.186
Male	18 (30.0)	42 (70.0)	
Female	23 (41.8)	32 (58.2)	
Age* (Year)			0.798
<60	13 (38.2)	21 (61.8)	
60-75	17 (32.7)	35 (67.3)	
>75	11 (39.3)	17 (60.7)	
Presence of other comorbidities			
Hyperblood pressure(HBP)	16 (27.1)	43 (72.9)	0.05
Cardiovascular disease	9 (36.0)	16 (64.0)	0.967
Presence of signs and symptoms*			
Fever	31 (43.1)	41 (56.9)	0.233
Cough	20 (39.2)	31 (60.8)	0.653
Asthenia	25 (41.7)	35 (58.3)	0.322
Myalgia	14 (43.8)	18 (56.3)	0.572
Dyspnea	23 (38.3)	37 (61.7)	0.409
CT or (scanner)*			0.220
Class I	12 (50.0)	12 (50.0)	
Class II	10 (41.7)	14 (58.3)	
Class III	8 (24.2)	25 (75.8)	
Class IV	2 (33.3)	4 (66.7)	
Complication and evolution			
Recovery			0.011
Yes	32 (44.4)	40 (55.6)	
No	9 (20.9)	34 (79.1)	
Intensive care unit			0.023
Yes	10 (22.7)	34 (77.3)	
No	31 (43.7)	40 (56.3)	
Death			0.022
Yes	8 (21.1)	30 (78.9)	
No	33 (42.9)	44 (57.1)	
Severity*			0.626
Yes	26 (36.1)	46 (63.9)	
No	10 (41.7)	14 (58.3)	

Comparison of clinical and biological values between the metformin group and the no-metformin group

The results of the physical examination and laboratory parameters in patients who used metformin and those who used alternative diabetes treatments are presented in Table 3.

**Table 3:** Comparison of clinical and biological value between the metformin group and no-metformin group LYM: lymohocyte; NEUT: neutrophils; PLT: platelet; PT: prothrombin time; ALT: alanine aminotransferase; SGOT: serum glutamic-oxaloacetic transaminase; GGT: gamma-glutamyl-transpeptidase; CRP: c-reactive protein *Data expressed as median and IQR; p-value<0.05 is significant.

Variables	N	With metformin	N	Without metformin	Normal range	P-value
Clinical parameters		Mean±SD		Mean±SD		
		Median (25;75)*		Median (25;75)*		
Oxygen saturation, %	36	85.9±11.9	60	82.8±13.7	95-100	0.264
Breathing rate, cpm	33	26.7±5.65	63	27.4±7.05	12-20	0.616
Diastolic, mmHg	38	70.24±10.19	65	70.34±10.61	80-89	0.752
Systolic, mmHg	38	130.3±10.91	65	130.5±20.22	120-139	0.744
Heart rate, bpm	39	84.7±16.4	65	89.5±17.2	60-100	0.166
Laboratory parameters						
Hemoglobin	39	11.9±2.05	63	11.9±2.7	11.5-15.5	0.953
Leukocyte, x10^3^/µL	39	10.558±4.9	63	13.196±9.1	4.0-10.0	0.097
LYM, x10^3^/µL	38	11.3±7.4	62	10.5±7.3	1.0-4.0	0.590
NEUT, x10^3^/µL	39	8.7±4.6	63	10.5±6.3	1.5-7	0.126
PLT, x10^3^/µL	39	250.6±108.4	64	271.1±121.2	150-400	0.390
D-dimer (ng/L)	34	1.53 (0.97-2.60)	56	2.12 (0.99-5.78)	<0.5	0.213
PT%	37	82.7±22.5	57	85.8±21.7	70-100	0.517
Fibrinogen (g/L)	34	6.07±2.46	49	5.92±2.06	2.0-4.0	0.762
Glycemia (g/L)	40	2.48±1.15	73	3.00±1.36	0.7-1.10	0.043
Urea (g/L)	40	0.435 (0.255-0.738)	74	0.665 (0.392-1.20)	0.15-0.55	0.007
Creatinine (mg/L)	41	9.20 (7.40-13.9)	73	11.8 (8.20-19.4)	5.7-12.5	0.037
SGPT or ALT (UI/L)	29	26.5 (21-44)	47	28 (17-43)	0-55	0.645
SGOT (UI/L)	34	40 (30-64.5)	58	34 (23.3-58.8)	5-34	0.423
GGT (U/L)	29	40 (27-93)	46	41 (24-60.5)	9-36	0.683
Alkaline reserve (mEq/L)	39	22.1±5.78	71	22.1±7.89	22-31	0.982
Sodium (mEq/L)	40	136±7.78	74	137±7.59	136-145	0.415
Chlore (mEq/L)	40	98.3±10.2	74	99±8.81	98-107	0.718
Total protein (g/L)	40	63.1±9.62	72	64.2±9.71	95-100	0.580
Ferritin (ng/mL)	33	554 (348-1079)	65	689 (385-1192)	21-274	0.274
CRP (mg/L)	40	166±114	71	148±109	<5	0.405

No significant differences were observed in the clinical parameters between the two groups of patients. The laboratory parameters showed a statistically significant difference between patients with and without metformin, specifically in glycemia, urea, and creatinine, with p-values of 0.043, 0.007, and 0.037, respectively.

Risk factors associated with in-hospital mortality in COVID-19 diabetic patients

In light of the significant differences observed in the aforementioned parameters between the groups, and given the variability of the dependent variable (mortality rate=33%), the factors selected for multivariable analysis were as follows: age, sex, glycemia, and the use or non-use of metformin. To eliminate and take better account of confounding and reporting biases, these parameters were controlled in the univariable analysis. The results of the multivariate logistic regression model indicated that age (OR=1.06; 95% CI (1.02-1.10) p=0.002) and glycemia (OR=1.49; 95% IC (1.05-2.11); p=0.024) were significantly associated with mortality in patients with T2DM and COVID-19. Conversely, after adjustments for age and glycemia, the use of metformin in type 2 diabetic patients with COVID-19 was identified as a protective factor (OR=0.34; 95% IC (0.12-0.95); p=0.041).

The results of the univariate and multivariate logistic regression analyses of risk factors associated with mortality in patients with T2DM and COVID-19 are presented in Table 4.

**Table 4:** Risk factors associated with mortality in COVID-19 diabetic patients with T2DM *Yes/No T2DM, type 2 diabetes Models were applied to 115 participants yielding 38 deaths (33%).

^Variable^	^Mortality (N=38)^					
	^Univariate^			^Multivariate^		
	^OR^	^95% IC^	^P-^^value^	^OR^	^95% IC^	^P-value ^
^Age (year)^	^1.05^	^(1.02-1.09) ^	^0.002 ^	^1.06 ^	^(1.02-1.10) ^	^0.002^
^Sex^	^1.20^	^(0.55-2.62)^	^0.641^	^0.87^	^(0.35-2.15)^	^0.777^
^Glycemia^	^1.60^	^(1.16-2.20) ^	^0.004^	^1.49^	^(1.05-2.11)^	^0.024^
^Use metformin*^	^0.356 ^	^(0.14-0.87)^	^0.024^	^0.34^	^(0.12-0.95)^	^0.041^

## Discussion

The main objective of this retrospective study was to ascertain whether the use of metformin in patients with T2DM who were hospitalized with a diagnosis of a SARS-CoV-2 infection was associated with a reduced risk of dying due to the illness.

The findings of our study indicate that patients treated with metformin exhibited superior glycemic control during their hospitalization compared to patients who were administered alternative antidiabetic agents. Similarly, Samuel et al. demonstrated the capacity of metformin to diminish blood glucose levels and enhance insulin stability, thereby reducing the likelihood of SARS-CoV-2 infections [18]. Furthermore, the findings of this study indicate that patients with diabetes who are undergoing metformin treatment exhibit decreased levels of urea and creatinine in comparison to those who are receiving treatment with an alternative antihyperglycemic medication. A recent cohort study conducted by Wang et al. demonstrated that patients with T2DM who were treated with metformin displayed superior renal outcomes, particularly in terms of creatinine levels [19]. Moreover, the study carried out by Song and colleagues has invested in evaluating the role of metformin as a promising pharmacological tool for renal diseases with or without T2DM considering the clinical application and long-term safety [20].

We discovered that no statistically significant difference was observed in the remaining biological and clinical parameters between the group that used metformin and the group that did not. Our findings align with those of Luo et al. who demonstrated no statistically significant difference in several biological and biochemical parameters between the metformin-treated group and those that did not receive metformin [12]. Conversely, the CORONADO study, conducted to examine the phenotypic characteristics and main outcomes of patients with diabetes who were hospitalized with confirmed SARS-CoV-2 between March 10 and April 10, 2020, and which included a larger sample size (1,317 patients), found differences in biological and clinical parameters between the groups of metformin users and non-users [11].

In addition, the findings of our study indicated that advanced age and high glycemia were associated with higher incidence mortality among patients with SARS-CoV2 infection. A study designed and conducted in England has shown that mortality in people with type 2 was associated not only with comorbidities such as cardiovascular and renal complications of diabetes but, independently, also with glycemic control and older age [2]. However, after adjusting for age and blood glucose, metformin remained a protective factor for mortality in adults with COVID-19. The observational study conducted in Wuhan, China, by Luo et al., concluded that hospital mortality was lower in patients with COVID-19 who were treated for diabetes with metformin than those who did not receive metformin [12]. Similarly, a systematic review and meta-analysis carried out in 2020 by Lukito et al. showed that metformin consumption was associated with lower mortality [21]. The retrospective study of Luo et al. reported that despite similar baseline patient characteristics (no significant difference in age, sex, clinical severity of COVID-19, and other associated comorbidities) and identical laboratory parameters between metformin users versus non-users, there were significantly fewer in-hospital mortality [12]. Furthermore, a retrospective, multicenter study involving a large sample was conducted in the USA, which indicated that the dose-dependent effect of metformin had a favorable impact on mortality rates and severity and seemed to decrease COVID-19-related hospitalization of diabetic patients [22].

In contrast, a retrospective study conducted by Goa and colleagues indicated that metformin therapy was related to an increased severity of SARS-CoV-2 infection and a higher number of life-threatening complications [23]; the outcomes of this investigation were likely attributable to two primary factors. First, the study’s modest size. Second, an absence of notable variations in clinical and biological characteristics between the groups.

In addition, a randomized trial was conducted by Bramante and colleagues on patients with COVID-19 and concluded that treatment with metformin does not prevent the occurrence of hypoxemia, emergency room visits, hospitalization, or death associated with COVID-19 [24]. Watson et al. asserted that the impact of metformin on the progression of SARS-CoV-2 infection may have significant clinical implications for managing diabetes during the disease. This is because metformin is typically discontinued in patients hospitalized for pneumonia or respiratory insufficiency due to the potential for increased risk of lactic acidosis [25].

Additionally, previous studies have well explained and demonstrated the crucial role of metformin treatment in the induction of the expression of the angiotensin-converting enzyme 2 (ACE2; which binds to the viral spike protein) via the activation of AMP-activated protein kinase pathways. This activation promotes the phosphorylation of the ACE2 receptor at serine 680 (SER680) thus causing the change in its conformation, which inhibits the binding of the SARS-COV2 spike protein to the ACE2. Consequently, the reduction of viral entry into the body's cells [26]. On the other hand, the regulation of the renin-angiotensin-aldosterone system (RAAS) is largely influenced by the ACE2 receptor; this system provides support and protection for cardiovascular, pulmonary, anti-inflammatory, and survival rates for patients diagnosed with SARS-CoV-2 infection [26]. These mechanisms offer biological plausibility to our findings and support the rigorous studies evaluating the impact of metformin [27-29].

Thus, the mechanisms of action of metformin that have been elucidated to date are of great importance and encompass many processes. These include the activation of AMPK in the liver by metformin, which results in the elimination of fatty acid synthesis and gluconeogenesis. Metformin activates AMPK in skeletal muscle, which increases the translocation of glucose transporter 4 to the cell membrane and thereby increases glucose uptake. Additionally, metformin suppresses glucagon signaling in the liver by inhibiting adenylate cyclase, which leads to the suppression of gluconeogenesis. Metformin can also suppress the mechanistic target of rapamycin (mTOR) by activating AMPK in pre-neoplastic cells, suppressing cell growth, and increasing apoptosis in pre-neoplastic cells [27]. Finally, metformin prevents severe kidney failure and vascular calcification via the AMPK signaling [30].

It is noteworthy that while the global dissemination of the SARS-CoV-2 virus has resulted in the declaration of a pandemic, reports have indicated that metformin utilization may be associated with decreased risk of mortality in individuals with both SARS-CoV-2 infection and T2DM mellitus. This finding offered a glimmer of hope amid the global health crisis [31].

## Conclusions

This study is the first of its kind in Morocco to assess the association between metformin use and mortality in type 2 diabetic patients with COVID-19. However, the results must be interpreted with consideration and caution. Nevertheless, valuable information has been obtained that will inform future research direction regarding this famous drug (metformin). Still, the limitations of this investigation should be noted. First, the limited number of diabetes samples; second, the retrospective nature of the study; and third, the lack of data in several patient records during this period of the pandemic. We recognize that our results cannot be generalized to the entire Moroccan population, but these findings rather highlight the need for further exploration and randomized trials in the future to more rigorously study the potential mechanisms of metformin that could lead to similar and confirmatory results. Furthermore, given the extensive use of metformin, it is imperative to conduct formal proof-based clinical trials across diverse ethnic and geographic populations to gain a deeper understanding of the potential association between metformin and complications associated with viral infections, particularly in the context of SARS-CoV-2. Our findings will contribute to and reinforce the findings of other international studies that have addressed this topic.

## References

[1] Tong ZW, Grant E, Gras S (2021). The role of T-cell immunity in COVID-19 severity amongst people living with type II diabetes. FEBS J.

[2] Holman N, Knighton P, Kar P (2020). Risk factors for COVID-19-related mortality in people with type 1 and type 2 diabetes in England: a population-based cohort study. Lancet Diabetes Endocrinol.

[3] Fang L, Karakiulakis G, Roth M (2020). Are patients with hypertension and diabetes mellitus at increased risk for COVID-19 infection?. Lancet Respir Med.

[4] Cao H, Baranova A, Wei X, Wang C, Zhang F (2023). Bidirectional causal associations between type 2 diabetes and COVID-19. J Med Virol.

[5] Wang J, Cooper JM, Gokhale K (2021). Association of metformin with susceptibility to COVID-19 in people with type 2 diabetes. J Clin Endocrinol Metab.

[6] Silverii GA, Fumagalli C, Rozzini R, Milani M, Mannucci E, Marchionni N (2024). Is metformin use associated with a more favorable Covid-19 course in people with diabetes?. J Clin Med.

[7] Erickson SM, Fenno SL, Barzilai N (2023). Metformin for treatment of acute COVID-19: systematic review of clinical trial data against SARS-CoV-2. Diabetes Care.

[8] Bramante CT, Ingraham NE, Murray TA (2021). Metformin and risk of mortality in patients hospitalised with COVID-19: a retrospective cohort analysis. Lancet Healthy Longev.

[9] Ma Z, Krishnamurthy M (2023). Is metformin use associated with low mortality in patients with type 2 diabetes mellitus hospitalized for COVID-19? a multivariable and propensity score-adjusted meta-analysis. PLoS One.

[10] Li Y, Yang X, Yan P, Sun T, Zeng Z, Li S (2021). Metformin in patients with COVID-19: a systematic review and meta-analysis. Front Med (Lausanne).

[11] Lalau JD, Al-Salameh A, Hadjadj S (2021). Metformin use is associated with a reduced risk of mortality in patients with diabetes hospitalised for COVID-19. Diabetes Metab.

[12] Luo P, Qiu L, Liu Y (2020). Metformin treatment was associated with decreased mortality in COVID-19 patients with diabetes in a retrospective analysis. Am J Trop Med Hyg.

[13] Cheng X, Xin S, Chen Y (2021). Effects of metformin, insulin on COVID-19 patients with pre-existed type 2 diabetes: a multicentral retrospective study. Life Sci.

[14] Bornstein SR, Rubino F, Khunti K (2020). Practical recommendations for the management of diabetes in patients with COVID-19. Lancet Diabetes Endocrinol.

[15] Singh AK, Singh R (2020). Is metformin ahead in the race as a repurposed host-directed therapy for patients with diabetes and COVID-19?. Diabetes Res Clin Pract.

[16] Sharma S, Ray A, Sadasivam B (2020). Metformin in COVID-19: a possible role beyond diabetes. Diabetes Res Clin Pract.

[17] Gallo Marin B, Aghagoli G, Lavine K (2021). Predictors of COVID-19 severity: a literature review. Rev Med Virol.

[18] Samuel SM, Varghese E, Büsselberg D (2021). Therapeutic potential of metformin in COVID-19: reasoning for its protective role. Trends Microbiol.

[19] Wang HH, Lin SH, Hung SY (2024). Renal protective effect of metformin in type 2 diabetes patients. J Clin Endocrinol Metab.

[20] Song A, Zhang C, Meng X (2021). Mechanism and application of metformin in kidney diseases: an update. Biomed Pharmacother.

[21] Lukito AA, Pranata R, Henrina J, Lim MA, Lawrensia S, Suastika K (2020). The effect of metformin consumption on mortality in hospitalized COVID-19 patients: a systematic review and meta-analysis. Diabetes Metab Syndr.

[22] Ghany R, Palacio A, Dawkins E (2021). Metformin is associated with lower hospitalizations, mortality and severe coronavirus infection among elderly medicare minority patients in 8 states in USA. Diabetes Metab Syndr.

[23] Gao Y, Liu T, Zhong W, Liu R, Zhou H, Huang W, Zhang W (2020). Risk of metformin in patients with type 2 diabetes with COVID-19: a preliminary retrospective report. Clin Transl Sci.

[24] Bramante CT, Huling JD, Tignanelli CJ (2022). Randomized trial of metformin, ivermectin, and fluvoxamine for COVID-19. N Engl J Med.

[25] Watson KE, Dhaliwal K, Robertshaw S (2023). Consensus recommendations for sick day medication guidance for people with diabetes, kidney, or cardiovascular disease: a modified Delphi process. Am J Kidney Dis.

[26] Malhotra A, Hepokoski M, McCowen KC, Shyy JY (2020). ACE2, metformin, and COVID-19. Iscience.

[27] Kaneto H, Kimura T, Obata A, Shimoda M, Kaku K (2021). Multifaceted mechanisms of action of metformin which have been unraveled one after another in the long history. Int J Mol Sci.

[28] Hariyanto TI, Kurniawan A (2020). Metformin use is associated with reduced mortality rate from coronavirus disease 2019 (COVID-19) infection. Obes Med.

[29] Wiernsperger N, Al-Salameh A, Cariou B, Lalau JD (2022). Protection by metformin against severe COVID-19: an in-depth mechanistic analysis. Diabetes Metab.

[30] Cao X, Li H, Tao H (2013). Metformin inhibits vascular calcification in female rat aortic smooth muscle cells via the AMPK-eNOS-NO pathway. Endocrinol.

[31] Zumla A, Hui DS, Azhar EI, Memish ZA, Maeurer M (2020). Reducing mortality from 2019-nCoV: host-directed therapies should be an option. Lancet.

